# Repeated, low-dose oral esketamine in patients with treatment-resistant depression: pilot study

**DOI:** 10.1192/bjo.2021.1059

**Published:** 2021-12-06

**Authors:** Sanne Y. Smith-Apeldoorn, Jolien K. E. Veraart, Henricus G. Ruhé, Marije aan het Rot, Jeanine Kamphuis, Marrit K. de Boer, Robert A. Schoevers

**Affiliations:** Department of Psychiatry, University of Groningen, University Medical Center Groningen, The Netherlands; Department of Psychiatry, University of Groningen, University Medical Center Groningen, The Netherlands; and Department of Mood Disorders, PsyQ Haaglanden, Parnassia Psychiatric Institute, The Netherlands; Department of Psychiatry, University of Groningen, University Medical Center Groningen, The Netherlands; and Department of Psychiatry, Radboud University Medical Center, The Netherlands; Department of Psychology, University of Groningen, The Netherlands; Department of Psychiatry, University of Groningen, University Medical Center Groningen, The Netherlands; Department of Psychiatry, University of Groningen, University Medical Center Groningen, The Netherlands; Department of Psychiatry, University of Groningen, University Medical Center Groningen, The Netherlands

**Keywords:** Esketamine, oral administration, treatment-resistant depression, tolerability, safety

## Abstract

**Background:**

Intravenous infusion of ketamine can produce rapid and large symptom reduction in patients with treatment-resistant depression (TRD) but presents major obstacles to clinical applicability, especially in community settings. Oral esketamine may be a promising addition to our TRD treatment armamentarium.

**Aims:**

To explore the safety, tolerability and potential clinical effectiveness of a 3-week treatment with repeated, low-dose oral esketamine.

**Method:**

Seven patients with chronic and severe TRD received 1.25 mg/kg generic oral esketamine daily, over 21 consecutive days. Scores on the Systematic Assessment for Treatment Emergent Events (SAFTEE), Community Assessment of Psychic Experiences (CAPE), Clinician Administered Dissociative States Scale (CADSS) and Hamilton Rating Scale for Depression (HRSD) instruments, as well as blood pressure and heart rate, were repeatedly assessed.

**Results:**

Treatment with oral esketamine was well-tolerated. No serious side-effects occurred, and none of the participants discontinued treatment prematurely. Psychotomimetic effects were the most frequently reported adverse events. Mean HDRS score decreased by 16.5%, from 23.6 to 19.7. Three participants showed reductions in HDRS scores above the minimum clinically important difference (eight-point change), of whom two showed partial response. No participants showed full response or remission.

**Conclusions:**

These results strengthen the idea that oral esketamine is a safe and well-tolerated treatment for patients with chronic and severe TRD, but therapeutic effects were modest. Results were used to design a randomised controlled trial that is currently in progress.

Depression is one of the most impactful conditions worldwilde in terms of individual suffering, lost productivity and healthcare costs.^1^ Unfortunately, response to treatment is often unsuccessful. Antidepressant medication and psychotherapy are ineffective in approximately 30% of patients.^2^ Although electroconvulsive therapy (ECT) is more effective, cognitive side-effects limit its use^3^ and there is a relatively high risk of relapse.^4^ Hence, there is a pressing need to develop new treatment strategies for depression generally, and for treatment-resistant depression (TRD) specifically.

## Ketamine for depression

Multiple lines of evidence support the idea that ketamine, an N-methyl-D-aspartate (NMDA) receptor antagonist, is an effective treatment for depression. A single intravenous infusion of a subanaesthetic dose can produce rapid and large symptom reduction in patients with unipolar and bipolar TRD, which generally lasts for about 1 week.^5^ These effects can be extended with repeated intravenous administration.^6,7^ However, this continuation procedure is relatively invasive and costly, and often brings about acute psychiatric (e.g. dissociation, anxiety, agitation) and somatic (e.g. headache, dizziness, cardiovascular) side-effects.^8^ These disadvantages present major obstacles to clinical use, especially in community settings.

To date, several studies have reported on the antidepressant properties of oral ketamine.^9,10^ They suggest that oral ketamine may also be effective in TRD, and that side-effects are well-tolerated. Moreover, chronic pain management data indicate that oral ketamine may safely be used for longer periods of time, including at home.^11^ Importantly, oral administration is associated with a lower risk of misuse compared with other routes of administration.^12^ Therefore, oral ketamine may be a suitable alternative for intravenous ketamine in the treatment of TRD.

## Esketamine

It should be noted that in most studies conducted to date, ketamine has been administered as a racemic mixture comprising its R-(–)enantiomer (arketamine) and S-(+)enantiomer (esketamine). Esketamine's NMDA receptor-binding affinity is three to four times higher than that of arketamine.^13^ It has been assumed that the majority of ketamine's antidepressant properties result from NMDA receptor antagonism and the consequent impact on glutamate neurotransmission.^14,15^ This would imply that esketamine could yield a better therapeutic effect than arketamine. Indeed, rapid and robust antidepressant effects of esketamine have been observed,^16^ and in 2019 an intranasal application of esketamine was approved by the USA Food and Drug Administration. However, the efficacy and safety of this esketamine nasal spray have been questioned,^17^ and the spray is currently expensive, limiting its widespread use.^18^ Comparably, oral (es)ketamine is widely available.

Given the advantages of oral over intravenous administration and indications for therapeutic efficacy of esketamine, oral esketamine may be a promising addition to treatment options for TRD. The aim of the present study was to explore the safety and tolerability of a 3-week treatment with oral esketamine in patients with chronic and severe TRD, and determine its potential clinical effectiveness.

## Method

### Sample

Participants were recruited at the Department of Psychiatry at the University Medical Center Groningen. We enrolled adults who met DSM-IV criteria for a major depressive episode (MDE). Additional inclusion criteria were severe symptom severity (score of >18 on the 17-item Hamilton Rating Scale for Depression (HRSD)) and TRD (insufficient response to four or more antidepressants or ECT during lifetime, given for ≥4 weeks in standard therapeutic doses). Exclusion criteria were psychotic features; past or current psychotic disorder; current substance dependence; primary diagnosis of personality disorder; and any contraindication for esketamine treatment, according to its Summary of Product Characteristics.

### Assessments

Safety and tolerability of treatment were assessed weekly by a staff medical doctor, using the Systematic Assessment for Treatment Emergent Events (SAFTEE), the Community Assessment of Psychic Experiences (CAPE) and the Clinician Administered Dissociative States Scale (CADSS). We assessed the occurrence of hypertension (systolic blood pressure > 160; diastolic blood pressure > 110) and tachycardia (beats per minute > 100) daily. A medical doctor examined change in depressive symptom severity, expressed as a change in total HRSD score between pre-treatment and end of treatment (3 weeks) and between end of treatment and follow-up (2 weeks).

### Treatment protocol

Participants were prescribed an oral solution containing 10 mg/mL generic esketamine three times a day, over 21 days. During the first 4 days, dosages were gradually increased (if well-tolerated) by 0.25 mg/kg per day, from 0.5 mg/kg per day up to a maximum of 1.25 mg/kg per day. The first doses were taken at the clinic and subsequent doses could be taken at home, depending on the participant's clinical state.

The maximum dose of 1.25 mg/kg per day was based on previous intravenous studies, most of which have used 0.5 mg/kg racemic ketamine. Given that 0.5 mg racemic ketamine includes 0.25 mg esketamine, esketamine accounts for 80% of the NMDA receptor antagonism of racemic ketamine^19^ and the bioavailability of oral esketamine is estimated to be around 20%,^20^ the maximum dose would be equivalent to 1.5 mg/kg. However, given potential additional pharmacodynamic properties of ketamine's metabolites^21^ and evidence that 1.25 mg/kg per day oral esketamine could be effective in TRD,^22^ we used a cautious maximum daily dose of 1.25 mg/kg per day to prevent overtreatment and associated side-effects. The daily dose was divided into three administrations, preventing high peak blood concentrations. This is expected to minimise acute side-effects, and is in line with ketamine use in the field of pain management, where there is ample experience with oral application of ketamine,^11^ and with several previous studies on oral ketamine in patients with depressive disorders.^23^

This treatment protocol was submitted to the Medical Ethics Review Board of the University Medical Center Groningen (approval number M17.217644), which exempted it from review under the Dutch Medical Research involving Human Subjects Act 1999. Our protocol was considered a compassionate use programme, allowing the use of an unauthorised medicine under strict conditions. Before providing written consent, participants received an oral and written explanation of the procedures, potential benefits and potential risks. Participants continued any antidepressant treatment they had been receiving before the start of esketamine, including psychotherapy.

### Data processing

The incidence of adverse events was calculated by comparing SAFTEE, CAPE and CADSS data during treatment and pre-treatment. Moderate discomforts (SAFTEE) were defined as an increase of ‘not present’ to ‘moderate’, or ‘mild’ to ‘severe’. Severe discomforts were defined as an increase of ‘not present’ to ‘severe’. The onset of new delusional thoughts (CAPE) or dissociative symptoms (CADSS) was defined as a change of ‘not present’ at baseline to ‘present’ during treatment, regardless of the severity. Increase of delusional thoughts or dissociative symptoms was defined as an increase of at least two levels of severity of pre-existing symptoms. We defined the minimum clinically important difference (MCID) as ≥8-point decrease in HRSD score,^24^ remission as an HRSD score ≤7, response as a ≥50% reduction in HRSD score and partial response as a ≥25% reduction in HRSD score. As pilot studies are not formally powered to assess effect, significance levels are not provided.

## Results

Six patients with unipolar depression and one patient with bipolar depression participated. On average, participants had used four antidepressant medications during the current MDE. All had received augmentation treatment during the current MDE, five had received ECT and four had also received psychotherapy. The average duration of the current MDE was 5.3 years (Tables 1 and 2). For case descriptions, see Supplementary material available at https://doi.org/10.1192/bjo.2021.1059.

**Table 1:** Sociodemographic and psychiatric characteristics (*N* = 7)

Characteristics	Mean (range) or *n* (%)
Sociodemographic	
Age, years	51.6 (36–80)
Female gender	4 (57%)
Psychiatric diagnosis and history	
Major depressive disorder	6 (86%)
Bipolar disorder	1 (14%)
Psychiatric comorbidities	3 (43%)
Age at onset of first depressive episode	30.7 (12–67)
Number of depressive episodes	4.0 (1–10)
Current depressive episode	
Severity, HRSD score	23.6 (17–34)
Duration, years	5.3 (2–13)
Number of antidepressant drug trials	4.3 (1–8)
Received augmentation treatment	7 (100%)
Received electroconvulsive therapy	5 (71%)
Received psychotherapy	4 (57%)

HRSD, Hamilton Rating Scale for Depression.

**Table 2:** Case presentation

Case	Gender	Age, years	Psychiatric diagnosis	Age at onset, years	Number of major depressive episodes	Duration of current episode, years	Current episode treatment history	Safety and tolerability	Efficacy (HRSD)				
									Before treatment	End of treatment	Δ (%)	Follow-up week 1	Follow-up week 2
1	Male	54	Bipolar depression	46	2	5	5 antidepressant trials; addition/augmentation of antidepressants/mood stabilisers/antipsychotics; ECT: yes	SAFTEE: drowsiness, fatigue, muscle cramps and stiffness, difficulty urinating, difficulty finding words; CAPE: thoughts of control and persecution; total score before treatment: 3; total score at end of treatment: 3	17	9	−8 (47)	14	16
2	Female	41	MDD, BPD, eating disorder	38	1	3	8 antidepressant trials; addition/augmentation of antidepressants/mood stabilisers/antipsychotics; ECT: no; other: psychotherapy	SAFTEE: abnormal sensations, slurred speech, nausea/vomiting, difficulty finding words, dizziness, potential (baseline score missing) sexual dysfunction; CAPE: thoughts of persecution; total score before treatment: 14; total score at end of treatment: 16; CADSS: dissociative symptoms; total score before treatment: 24; total score at end of treatment: 22	34	26	−8 (24)	32	38
3	Female	45	MDD, PTSD, ADHD	12	−	2	3 antidepressant trials; addition/augmentation of antidepressants/mood stabilisers/antipsychotics; ECT: yes	SAFTEE: fluid retention, weight gain, hot flashes, nausea/vomiting, diarrhoea, joint pain; CADSS: dissociative symptoms; total score before treatment: 1; total score at end of treatment: 2	27	26	−1 (4)	26	25
4	Female	41	MDD, personality disorder	26	5	8	5 antidepressant trials; addition/augmentation of antidepressants/mood stabilisers/antipsychotics; ECT: yes; other: psychotherapy	SAFTEE: drowsiness, fatigue, dizziness, excessively sweating, sexual dysfunction	19	10	−9 (47)	−	−
5	Male	36	MDD	14	10	2	2 antidepressant trials; addition/augmentation of antidepressants/mood stabilisers/antipsychotics; ECT: yes	SAFTEE: nightmares, feeling unreal, headache, potential (baseline scores missing) nausea/vomiting, abdominal discomfort, constipation, decreased appetite, weight loss, mental decline, apathy; CADSS: dissociative symptoms; total score before treatment: 1; total score at end of treatment: 0	20	21	+1 (5)	−	−
6	Male	80	MDD	67	1	13	6 antidepressant trials; addition/augmentation of antidepressants/mood stabilisers/antipsychotics; ECT: yes; other: psychotherapy	SAFTEE: hallucinations, blurred vision, slurred speech, drowsiness, numbness, ringing in ears, abnormal sensations, potential (baseline scores missing) frequent urinating, sexual dysfunction, decreased appetite, increased appetite, difficulty finding words, emotional indifference, hot flashes, strange taste; CAPE: thoughts of control and persecution; total score before treatment: 15; total score at end of treatment: 5; CADSS: dissociative symptoms; total score before treatment: 37; total score at end of treatment: 41	24	22	−2 (8)	17	22
7	Female	64	MDD	12	5	4	1 antidepressant trial; addition/augmentation of antidepressants/mood stabilisers/antipsychotics; ECT: no; other: psychotherapy, chronotherapy	SAFTEE: muscle cramps and stiffness, sexual dysfunction	24	24	0 (0)	25	26

Δ indicates the difference in HDRS scores between before and after treatment. HRSD, Hamilton Rating Scale for Depression; ECT, electroconvulsive therapy; SAFTEE, Systematic Assessment for Treatment Emergent Events; CAPE, Community Assessment of Psychic Experiences; MDD, major depressive disorder; BPD, borderline personality disorder; CADSS, Clinician Administered Dissociative States Scale; PTSD, post-traumatic stress disorder; ADHD, attention-deficit hyperactivity disorder.

Treatment was well-tolerated overall. Although one participant requested dose reduction because of adverse events (headache, dissociation, emotional imbalance and heart palpitations), no participants discontinued treatment prematurely and no serious adverse events occurred. No participants reported ketamine cravings or an urge to use ketamine beyond the prescribed treatment period.

Onset of moderate discomforts or moderate increase of pre-existing discomforts was reported 45 times, and mostly involved sexual dysfunction (*n* = 3), difficulty finding words (*n* = 3) and nausea/vomiting (*n* = 3). Onset of severe discomforts was reported four times, and included sexual dysfunction, drowsiness, numbness and tinnitus. Of those reported, 19% of discomforts were reported after 1 week of treatment, 44% after 2 weeks and 38% after 3 weeks. Most discomforts were self-limiting before the end of follow-up, except for moderate drowsiness and fatigue in one participant, and (subjective) moderate fluid retention and hot flashes in another participant. Onset or increase of delusional thoughts was reported four times, and included thoughts of control and persecution. Onset or increase of dissociative symptoms was reported seven times. Both were mild and self-limiting. Hypertension or tachycardia did not occur.

**Fig. 1:**
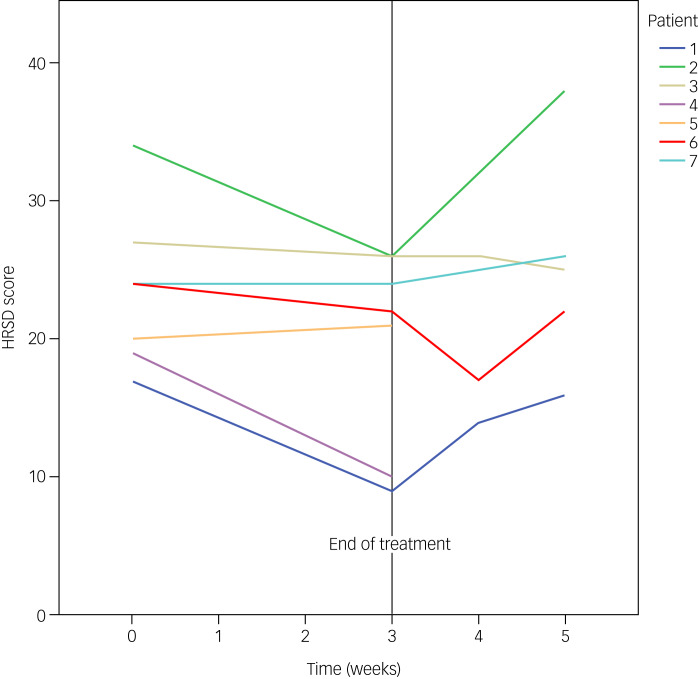
Hamilton Rating Scale for Depression (HRSD) score changes of each participant, shown as an individual line between baseline (week 0) and end of treatment (week 3), and between end of treatment (week 3) and follow-up (weeks 4 and 5). Change from baseline to the end of treatment: patient 1, −8 (−47%); patient 2, −8 (−24%); patient 3, −1 (−4%); patient 4, −9 (−47%); patient 5, +1 (+5%); patient 6, −2 (−8%); patient 7, 0 (0%).

All participants reported positive effects, including reduction of suicidal thoughts, improved mood and increased energy levels. The mean HRSD score decreased from 23.6 at baseline to 19.7 at week 3 (−16.5%). Three participants showed absolute reductions above MCID, of whom two showed partial response (Fig. 1). No participants showed full response or remission within the treatment period.

During follow-up, the mean HRSD score increased to 22.8 within 1 week and to 25.4 within 2 weeks. Two participants showed further reduction in HRSD scores during follow-up; however, three participants showed an increase in HRSD scores. Follow-up data for two participants are missing.

## Discussion

This pilot study was conducted to explore the safety, tolerability and potential clinical effectiveness of a 3-week treatment with repeated, low-dose generic oral esketamine. Treatment appeared safe and was well-tolerated. Most adverse events were moderate and self-limiting. Four adverse events had not resolved before the end of follow-up, namely drowsiness and fatigue in one patient and (subjective) fluid retention and hot flashes in another. It is unclear if these symptoms were linked to the esketamine treatment.

Although side-effects are common during treatment with (es)ketamine, little is known about the side-effects of repeated dosing, including possible cumulative and longer-term effects.^8^ (Es)ketamine-induced ulcerative cystitis and dependence are of particular concern.^25^ Potential tolerance to the drug cannot be ignored, particularly with daily dosing. Preclinical studies have demonstrated a significant escalation of intravenous (es)ketamine intake during repeated self-administration sessions in rats.^26,27^ However, tolerance to daily oral ketamine treatment in patients with chronic pain is not often observed.^11,28^ This may be because the oral route of administration is associated with a lower risk of misuse in general,^12^ and that after oral intake, the main metabolite norketamine reaches higher plasma levels as a result of extensive first-pass metabolism, and might have a more favourable safety profile than ketamine.^29^ Still, when prescribing oral (es)ketamine for patients with depression, the physician is expected to determine the possible risk/benefit ratio for every individual patient. In addition, after initial response, tapering ought to be the principle to limit the risk of long-term side-effects. Taking into account the possible risk of adverse events, we argue that initiation of (es)ketamine treatment should take place in an in-patient or day care setting, and follow-up treatment should be carefully monitored.

The therapeutic effect of oral esketamine in this study was modest, at least compared with that of intravenous esketamine^16^ and racemic ketamine.^5^ Although three out of seven participants reached MCID at the end of treatment, no participants showed full response or remission. This might be because our participants are considered among the most difficult to treat. All patients had severe and chronic TRD and high levels of treatment refractoriness, including for ECT. This predicts a poor response to any subsequent treatment. Therefore, we argue that any clinical benefit in this patient category is a major gain. Moreover, our 3-week treatment duration might have been too short to experience esketamine's full antidepressant effect. Longer treatment could have led to further improvement, underscored by the lack of plateaus in Fig. 1 (specifically, see cases 1, 2, 4 and 6). This would be in line with the findings of Jafarinia et al,^30^ showing a significant treatment effect of oral ketamine versus placebo after 6, but not 3 weeks of treatment.

Another potential explanation for the modest therapeutic effect can be found in the treatment regimen. Although first-pass metabolism was taken into account when determining the daily dose, it cannot be ruled out that blood levels of esketamine were insufficient for optimal treatment efficacy. We assumed a bioavailability of oral esketamine of 20%. However, lower bioavailability has also been reported.^31^ Consequently, 1.25 mg/kg per day could well be lower than the required dose for optimal antidepressant effects. Further, daily doses were divided into three administrations a day. Although this was done to reduce the risk of side-effects, peak esketamine blood levels needed to trigger a pharmacodynamical cascade may not have been reached. Unfortunately systematic blood levels of esketamine were not determined. At the same time, there is no good support for the idea that peak blood levels are preferable to more steady blood levels, and antidepressant effects of very low doses of sublingual ketamine have been described.^32^ We also note that although three non-responders received intravenous esketamine after completing the pilot, again this did not lead to a response (reduction in severity of symptoms of 0%, 2% and 14%; additional data available on request). This argues against the idea that insufficient blood levels unambiguously explain non-response.

A fourth potential explanation for the modest therapeutic effect is the use of the S-(+)enantiomer. Although it has long been assumed that the majority of ketamine's antidepressant properties stem from its impact on glutamate neurotransmission through NMDA receptor binding, the concept of NMDA receptor antagonism has been challenged, and various other molecular insights have been gained in the mechanistic pathways of ketamine and its enantiomers.^33^ This has recently renewed interest into alternatives like arketamine,^34^ and points to a need to directly compare the effects of racemic ketamine and its enantiomers in patients with depression.

This pilot study has several limitations. Inherent to the design, the study lacked a control group, blinding and randomisation. Further, our sample size was small and heterogeneous, with high levels of treatment resistance compared with other trials in the literature. In addition, as previously mentioned, blood levels of esketamine and its metabolites could have provided insight into the cause of the modest therapeutic effects in our patients, and should ideally be included in future studies. Despite these limitations, our results indicate that daily oral esketamine over a treatment duration of 3 weeks is safe and well-tolerated, and that, although the therapeutic effect was modest, it may provide relief from TRD, even in patients with a very poor prognosis. Based on these findings, we decided to design a randomised controlled trial with a treatment duration of 6 weeks and monitoring of blood levels of (nor)ketamine.^35^ If safety, tolerability and effectiveness are confirmed, oral esketamine could become a suitable treatment strategy for TRD.

## Data Availability

The data that support the findings of this study are available from the corresponding author, S.Y.S.-A., upon reasonable request.

## Supplementary material

For supplementary material accompanying this paper visit http://dx.doi.org/10.1192/bjo.2021.1059. click here to view supplementary material

## References

[1] Ferrari AJ, Charlson FJ, Norman RE, Patten SB, Freedman G, Murray CJL, Burden of depressive disorders by country, sex, age, and year: findings from the Global Burden of Disease Study 2010. PLoS Med 2013; 2013(10): e1001547. 10.1371/journal.pmed.1001547 PMC3818162 24223526

[2] Rush AJ, Trivedi MH, Wisniewski SR, Nierenberg AA, Stewart JW, Warden D, Acute and longer-term outcomes in depressed outpatients requiring one or several treatment steps: a STAR*D report. Am J Psychiatry 2006; 163: 1905–17. 17074942 10.1176/ajp.2006.163.11.1905

[3] Sackeim H, Prudic J, Fuller R, Keilp J, Lavori PW, Olfson M. The cognitive effects of ECT in community settings. Neuropsychopharmocology 2007; 32: 244–54. 10.1038/sj.npp.1301180 16936712

[4] Jelovac A, Kolshus E, McLoughlin DM. Relapse following successful electroconvulsive therapy for major depression: a meta-analysis. Neuropsychopharmacology 2013; 38: 2467–74. 23774532 10.1038/npp.2013.149 PMC3799066

[5] Kishimoto T, Chawla JM, Hagi K, Zarate CA, Kane JM, Bauer M, Single-dose infusion ketamine and non-ketamine N-methyl-D-aspartate receptor antagonists for unipolar and bipolar depression: a meta-analysis of efficacy, safety and time trajectories. Psychol Med 2016; 46: 1459–72. 26867988 10.1017/S0033291716000064 PMC5116384

[6] Aan het Rot M, Collins KA, Murrough JW, Perez AM, Reich DL, Charney DS, Safety and efficacy of repeated-dose intravenous ketamine for treatment-resistant depression. Biol Psychiatry 2010; 67: 139–45. 19897179 10.1016/j.biopsych.2009.08.038

[7] Kryst J, Kawalec P, Mitoraj AM, Pilc A, Brzostek T. Efficacy of single and repeated administration of ketamine in unipolar and bipolar depression: a meta-analysis of randomized clinical trials. Pharmacol Rep 2020; 72: 543–62. 32301056 10.1007/s43440-020-00097-z PMC7329804

[8] Short B, Fong J, Gálvez V, Shelker W, Loo CK. Side-effects associated with ketamine use in depression: a systematic review. Lancet Psychiatry 2018; 5: 65–78. 28757132 10.1016/S2215-0366(17)30272-9

[9] Rosenblat JD, Carvalho AF, Li M, Lee Y, Subramanieapillai M, McIntyre RS. Oral ketamine for depression: a systematic review. J Clin Psychiatry 2019; 80: 18r12475. 10.4088/JCP.18r12475 30995364

[10] Nuñez NA, Joseph B, Pahwa M, Seshadri A, Prokop LJ, Kung S, An update on the efficacy and tolerability of oral ketamine for major depression: a systematic review and meta-analysis. Psychopharmacol Bull 2020; 50: 137–63. 33012876 10.64719/pb.4376 PMC7511150

[11] Schoevers RA, Chaves TV, Balukova SM, Aan het Rot M, Kortekaas R. Oral ketamine for the treatment of pain and treatment-resistant depression. Br J Psychiatry 2016; 208: 108–13. 26834167 10.1192/bjp.bp.115.165498

[12] Farré M, Camí J. Pharmacokinetic considerations in abuse liability evaluation. Br J Addict 1991; 86: 1601–6. 1786493 10.1111/j.1360-0443.1991.tb01754.x

[13] Kohrs R, Durieux ME. Ketamine: teaching an old drug new tricks. Anesth Analg 1998; 87: 1186–93. 9806706 10.1097/00000539-199811000-00039

[14] Abdallah CG, Sanacora G, Duman RS, Krystal JH. The neurobiology of depression, ketamine and rapid-acting antidepressants: is it glutamate inhibition or activation? Pharmacol Ther 2018; 190: 148–58. 29803629 10.1016/j.pharmthera.2018.05.010 PMC6165688

[15] McIntyre RS, Rosenblat JD, Nemeroff CB, Sanacora G, Murrough JW, Berk M, Synthesizing the evidence for ketamine and esketamine in treatment-resistant depression: an international expert opinion on the available evidence and implementation. Am J Psychiatry 2021; 178: 383–99. 33726522 10.1176/appi.ajp.2020.20081251 PMC9635017

[16] Singh JB, Fedgchin M, Daly E, Xi L, Melman C, De Bruecker G, Intravenous esketamine in adult treatment-resistant depression: a double-blind, double-randomization, placebo-controlled study. Biol Psychiatry 2016; 80: 424–31. 26707087 10.1016/j.biopsych.2015.10.018

[17] Schatzberg F. A word to the wise about intranasal esketamine. Am J Psychiatry 2019; 176: 422–4. 31109197 10.1176/appi.ajp.2019.19040423

[18] Ross EL, Soeteman DI. Cost-effectiveness of esketamine nasal spray for patients with treatment-resistant depression in the United States. Psychiatr Serv 2020; 71: 988–97. 32631129 10.1176/appi.ps.201900625 PMC7920520

[19] Moaddel R, Abdrakhmanova G, Kozak J, Jozwiak K, Toll L, Jimenez L, Sub-anesthetic concentrations of (R,S)-ketamine metabolites inhibit acetylcholine-evoked currents in α7 nicotinic acetylcholine receptors. Eur J Pharmacol 2013; 698: 228–34. 23183107 10.1016/j.ejphar.2012.11.023 PMC3534778

[20] Chong CC, Schug SA, Page-Sharp M, Ilett KF. Bioavailability of ketamine after oral or sublingual administration. Pain Medicine 2006; 7: 469.

[21] Yang C, Kobayashi S, Nakao K, Dong C, Han M, Qu Y, AMPA receptor activation-independent antidepressant actions of ketamine metabolite (S)-norketamine. Biol Psychiatry 2018; 84: 591–600. 29945718 10.1016/j.biopsych.2018.05.007

[22] Paslakis G, Gilles M, Meyer-Lindenberg A, Deuschle M. Oral administration of the NMDA receptor antagonist S-ketamine as add-on therapy of depression: a case series. Pharmacopsychiatry 2010; 43: 33–5. 20013614 10.1055/s-0029-1237375

[23] Arabzadeh S, Hakkikazazi E, Shahmansouri N, Tafakhori A, Ghajar A, Jafarinia M, Does oral administration of ketamine accelerate response to treatment in major depressive disorder? Results of a double-blind controlled trial. J Affect Disord 2018; 235: 236-41. 10.1016/j.jad.2018.02.056 29660637

[24] Bobo WV, Angleró GC, Jenkins G, Hall-Flavin DK, Weinshilboum R, Biernacka JM. Validation of the 17-item Hamilton Depression Rating Scale definition of response for adults with major depressive disorder using equipercentile linking to Clinical Global Impression scale ratings: analysis of pharmacogenomic research network antidepressant medication pharmacogenomic study (PGRN-AMPS) data. Hum Psychopharmacol 2016; 31: 185–92. 26999588 10.1002/hup.2526 PMC5008690

[25] Morgan CJA, Curran HV. Independent Scientific Committee on Drugs. Ketamine use: a review. Addiction 2012; 107: 27–38. 21777321 10.1111/j.1360-0443.2011.03576.x

[26] Bonaventura J, Lam S, Carlton M, Boehm MA, Gomez JL, Solís O, Pharmacological and behavioral divergence of ketamine enantiomers: implications for abuse liability. Mol Psychiatry [Epub ahead of print] 15 Apr 2021. Available from: 10.1038/s41380-021-01093-2. PMC8517038 33859356

[27] De Luca MT, Badiani A. Ketamine self-administration in the rat: evidence for a critical role of setting. Pharmacology 2011; 214: 549–56. 10.1007/s00213-010-2062-x 21069515

[28] Blonk MI, Koder BG, Van den Bemt PMLA, Huygen FJPM. Use of oral ketamine in chronic pain management: a review. Eur J Pain 2010; 14: 466–72. 19879174 10.1016/j.ejpain.2009.09.005

[29] Holtman Jr. JR, Crooks PA, Johnson-Hardy JK, Hojomat M, Kleven M, Wala EP. Effects of norketamine enantiomers in rodent models of persistent pain. Pharmacol Biochem Behav 2008; 90: 676–85. 18586315 10.1016/j.pbb.2008.05.011

[30] Jafarinia M, Afarideh M, Tafakhori A, Arbabi M, Ghajar A, Noorbala AA, Efficacy and safety of oral ketamine versus diclofenac to alleviate mild to moderate depression in chronic pain patients: a double-blind, randomized controlled trial. J Affect Disord 2016; 204: 1–8. 27317968 10.1016/j.jad.2016.05.076

[31] Fanta S, Kinnunen M, Backman JT, Kalso E. Population pharmacokinetics of S-ketamine and norketamine in healthy volunteers after intravenous and oral dosing. Eur J Clin Pharmacol 2015; 71: 441–7. 25724645 10.1007/s00228-015-1826-y

[32] Lara DR, Bisol LW, Munari LR. Antidepressant, mood stabilizing and procognitive effects of very low dose sublingual ketamine in refractory unipolar and bipolar depression. Int J Neuropsychopharmacol 2013; 16: 2111–7. 23683309 10.1017/S1461145713000485

[33] Jelen LA, Young AH, Stone JM. Ketamine: a tale of two enantiomers. J Psychopharmacol 2021; 35: 109–23. 33155503 10.1177/0269881120959644 PMC7859674

[34] Leal GC, Bandeira ID, Correia-Melo FS, Telles M, Mello RP, Vieira F, Intravenous arketamine for treatment-resistant depression: open-label pilot study. Eur Arch Psychiatry Clin Neurosci 2021; 271: 577–82. 32078034 10.1007/s00406-020-01110-5

[35] Smith-Apeldoorn SY, Veraart JKE, Kamphuis J, Van Asselt ADI, Touw DJ, Aan het Rot M, Oral esketamine for treatment-resistant depression: rationale and design of a randomized controlled trial. BMC Psychiatry 2019; 19: 375–83. 31783823 10.1186/s12888-019-2359-1 PMC6884875

