# Supplementary material for: Repeated, low-dose oral esketamine in patients with treatment-resistant depression: pilot study
Source: BJPsych Open. 2021 Dec 6;8(1):e4. doi: 10.1192/bjo.2021.1059 (PMC8693908; doi:10.1192/bjo.2021.1059)
Supplement: Supplementary file 1 [file S2056472421010590sup001.pdf]

Repeated, low-dose oral esketamine in patients with treatment-resistant depression: a pilot study

Supplementary material: Case presentation

Case 1 was a 54-year-old man who was diagnosed with a bipolar disorder 8 years before, and had been chronically depressed for 5 years. He was also diagnosed with hypothyroidism and morbid obesity. During the current major depressive episode (MDE) he had failed to respond to 5 subsequent antidepressants (including a selective serotonin reuptake inhibitor (SSRI), a serotonin-norepinephrine reuptake inhibitor (SNRI), tricyclic antidepressants (TCA's), and a monoamine oxidase inhibitor (MAO-I)), augmentation of mood stabilizers, and electroconvulsive therapy (ECT). Esketamine (120 kg x 1.25 mg/kg = 150 mg/day) was given as add-on to clomipramine, lithium, and olanzapine.

Tolerability and safety: During treatment, the Systematic Assessment for Treatment Emergent Events (SAFTEE) showed the onset of moderate drowsiness, fatigue, muscle cramps and stiffness, difficulty urinating, and difficulty in finding words. Most adverse events were self-limiting before the end of follow-up, except for drowsiness and fatigue. The Community Assessment of Psychic Experiences (CAPE) showed the onset of thoughts of persecution (item 6, 7, and 10) and control (item 24), and a moderate increase on item 17 on (pre-existing) thoughts of control, although at the end and after treatment the patient reported that he had never experienced such thoughts. The Clinician Administered Dissociative States Scale (CADSS) showed no onset of new or increase of pre-existing dissociative symptoms. No hypertension, tachycardia, or serious treatment-emergent adverse events occurred.

Efficacy: During treatment, the Hamilton Depression Rating Scale – 17 items ($HDRS_{17}$) score decreased from 17 (moderate) at baseline to 9 (mild) at the end of treatment (47% decrease). After treatment, the $HDRS_{17}$ score increased to 14 (moderate) within 1 week and to 16 (moderate) within 2 weeks. At the end of treatment the patient reported that his 'depressed mood was not as deep as before', his 'interest was increased', and his 'concentration and sleep had improved'.

Case 2 was a 41-year-old woman who was diagnosed with a major depressive disorder (MDD) 3 years before, and had been chronically depressed since then. She was also diagnosed with borderline personality disorder, eating disorder, and post-operative foot pain. She had failed to respond to 8 subsequent antidepressants (including SSRI, SNRI, TCA and MAO-I), lithium and quetiapine augmentation, and 4 types of psychotherapy. Esketamine (70 mg/day) was given as add-on to bupropion, quetiapine, and psychotherapy.

Tolerability and safety: During treatment, the SAFTEE showed the onset of moderate abnormal sensations, slurred speech, nausea / vomiting, difficulty in finding words, and dizziness. All were self-limiting. The SAFTEE also showed the potential onset or increase of sexual dysfunction (baseline score missing). The CAPE showed no onset of new psychotic symptoms, but did show a moderate increase on item 7 on (pre-existing) persecution. The CADSS showed the onset of some new and an increase of some pre-existing dissociative symptoms. The total CADSS score however decreased from 24 at baseline to 22 at the end of treatment. No hypertension, tachycardia, or serious treatment-emergent adverse events occurred.

Efficacy: During treatment, the $HDRS_{17}$ score decreased from 34 (very severe) at baseline to 26 (very severe) at the end of treatment (24% decrease). After treatment, the $HDRS_{17}$ score increased to 32 (very severe) within 1 week and to 38 (very severe) within 2 weeks. At the end of treatment

the patient reported that she ‘experienced less emotional blunting’, ‘became able to cry’, and ‘experienced more depth in psychotherapy’. Anorexic thoughts had faded.

Case 3 was a 45-year-old woman with MDD that had been present since childhood, who had been chronically depressed for 2 years. She was also diagnosed with post-traumatic stress disorder, attention deficit hyperactivity disorder, and former cannabis use disorder. During the current MDE she had failed to respond to 3 subsequent antidepressants (SSRI, SNRI, and TCA), lithium augmentation, and ECT. Previously she had failed to respond to 5 antidepressants (TCA’s and MAO-I) and psychotherapy. Esketamine (75 mg/day) was given as add-on to citalopram.
Tolerability and safety: During treatment, the SAFTEE showed the onset of moderate fluid retention, weight gain, and hot flashes, and a moderate increase of pre-existing nausea / vomiting, diarrhea, and joint pain. Besides, the patient spontaneously reported headache, balance issues, and an itching skin. Most adverse events were self-limiting before the end of follow-up, except for fluid retention and hot flashes. The CAPE showed no onset of new or increase of pre-existing psychotic symptoms. The CADSS showed the onset of some new dissociative symptoms. The total CADSS score increased from 1 at baseline to 2 at the end of treatment. After treatment the CADSS score decreased to 0 within 1 week. No hypertension, tachycardia, or serious treatment-emergent adverse events occurred.
Efficacy: During treatment, the $HDRS_{17}$ score decreased from 27 (very severe) at baseline to 26 (very severe) at the end of treatment (4% decrease). After treatment, the $HDRS_{17}$ score remained constant within the first week, and further decreased to 25 (very severe) within 2 weeks (7% decrease). Interesting, at the end of treatment the patient reported that she ‘felt less depressed and anxious’ and ‘had less thoughts of dead’.

Case 4 was a 41-year-old woman who was diagnosed with MDD 15 years before, and had been chronically depressed for 8 years. She was also diagnosed with personality disorder, hypothyroidism, and pernicious anemia. During the current MDE she had failed to respond to 5 subsequent antidepressants (including SSRI, SNRI, TCA and MAO-I), lithium augmentation, ECT, and psychotherapy. Esketamine (82.5 mg/day) was given as add-on to paroxetine and quetiapine.

Tolerability and safety: During the tapering-in process a treatment protocol deviation occurred, with the consequence that the patient was twice prescribed 100 mg esketamine. She mainly reported acute but short-lasting visual hallucinations, nausea, and dizziness. During treatment, the SAFTEE showed the onset of moderate drowsiness, fatigue, dizziness, and excessively sweating, and the onset of severe sexual dysfunction. Follow-up data are missing. CAPE data, CADSS data, and data on blood pressure and heart rate are missing. No serious treatment-emergent adverse events occurred.

Efficacy: During treatment, the $HDRS_{17}$ score decreased from 19 (severe) at baseline to 10 (mild) at the end of treatment (47% decrease). After treatment, the depressive symptoms increased within 1 week according to the physician of the patient. Follow-up measurement data are missing. At the end of treatment the patient reported that her ‘mood had clearly improved’, and her ‘suicidal thoughts had partly faded’.

Case 5 was a 36-year-old man who was diagnosed with MDD 22 years before, and had been chronically depressed for 2 years. During the current MDE he had failed to respond to 2 subsequent antidepressants (TCA’s), lithium augmentation, and ECT. Previously he had failed to respond to 3 antidepressants (SSRI’s and SNRI) and psychotherapy. Upon study entry he had

refused a MAO-I. During esketamine treatment (90 mg/day), he did not receive any other depression treatment.

Tolerability and safety: During treatment, the SAFTEE showed the onset of moderate nightmares and of feeling unreal. The SAFTEE also showed moderate increase of pre-existing headache, and the potential onset or increase (baseline scores missing) of nausea / vomiting, abdominal discomfort, constipation, decreased appetite, weight loss, mental decline, and apathy. All adverse events were self-limiting. The CAPE showed no onset of new or increase of pre-existing psychotic symptoms. The CADSS showed the onset of some new and an increase of pre-existing dissociative symptoms in the first week of treatment. From the third week of treatment the CADSS showed no more dissociative symptoms at all. No hypertension, tachycardia, or serious treatment-emergent adverse events occurred.

Efficacy: During treatment, the $HDRS_{17}$ score increased from 20 (severe) at baseline to 21 (severe) after 3 weeks of treatment (5% increase). Because the patient reported that he 'experienced decrease of restlessness, worrying and emptiness', treatment was continued for 2 more weeks. The $HDRS_{17}$ score further increased from 21 after 3 weeks of treatment to 23 (very severe) at the end of treatment (10% increase).

Case 6 was an 80-year-old man who was diagnosed with MDD 13 years before, and had been chronically depressed since then. He had a medical history of cerebral infarction (2005). The patient had failed to respond to 6 subsequent antidepressants (including SSRI, SNRI, TCA and MAO-I), lithium and antipsychotics augmentation, ECT, and psychotherapy. Esketamine (90 mg/day) was given as add-on to nortriptyline, quetiapine and in-clinic day treatment.

Tolerability and safety: During treatment, the SAFTEE showed the onset of moderate hallucinations, blurred vision, and slurred speech, and the onset of severe drowsiness, numbness,

and ringing in ears. The SAFTEE also showed moderate increase of pre-existing abnormal sensations, and the potential onset or increase (baseline scores missing) of frequent urinating, sexual dysfunction, decreased ánd increased appetite, difficulty in finding words, emotional indifference, hot flashes, and a strange taste. All adverse events were self-limiting before the end of follow-up, except for the potential adverse event sexual dysfunction. The CAPE showed the onset of thoughts of control (item 15) and possibly of persecution (item 2; baseline score missing), although at the end of treatment the patient reported that he had never experienced such thoughts. The CADSS showed the onset of some new and an increase of some pre-existing dissociative symptoms. The total CADSS score increased from 37 at baseline to 41 at the end of treatment. After treatment the CADSS score decreased to 9 within 1 week. Data on blood pressure and heart rate are missing. No serious treatment-emergent adverse events occurred.
Efficacy: During treatment, the $HDRS_{17}$ score decreased from 24 (very severe) at baseline to 22 (severe) at the end of treatment (8% decrease). After treatment, the $HDRS_{17}$ score further decreased to 17 (moderate) within 1 week (29% decrease), and then increased to 22 (severe) within 1 week. At the end of treatment the patient's wife reported that the patient 'took more initiatives' and 'became more assertive'.

Case 7 was a 64-year-old woman with MDD that had been present since childhood, who had been chronically depressed for 4 years. She was also diagnosed with osteoarthritis, osteoporosis, and fibromyalgia. During the current MDE she had failed to respond to an SSRI, lithium augmentation, chronotherapy, day treatment, and psychotherapy. Previously she had failed to respond to 5 antidepressants (including SSRI, SNRI, and TCA) and several types of psychotherapy. Upon study entry she had refused ECT. During esketamine treatment (90 - 102.5 mg/day), she did not receive any other depression treatment.

Tolerability and safety: During treatment, the SAFTEE showed the onset of moderate muscle cramps and stiffness, and a moderate increase of pre-existing sexual dysfunction. Muscle cramps and stiffness were self-limiting. The CAPE and CADSS showed no onset of new or increase of pre-existing psychotic symptoms or dissociative symptoms. However, the patient did spontaneously report unacceptable headaches, dissociation (‘feeling high’), emotional imbalance and heart palpitations during treatment with 102.5 mg oral esketamine per day. After downward titration to 90 mg/day, short-lasting and mild dissociations remained. No hypertension, tachycardia, or serious treatment-emergent adverse events occurred.

Efficacy: The $HDRS_{17}$ baseline score of 24 (very severe) did not decrease nor increase during treatment. After treatment, the $HDRS_{17}$ score further increased to 25 (very severe) within 1 week, and to 26 (very severe) within 2 weeks. In contrast, at the end of treatment the patient reported that her ‘mood was improved’, her ‘interest was increased’ and that she was ‘more looking outwards’.
